# The Effect of Soil-Structure Interaction on the Seismic Response of Structures Using Machine Learning, Finite Element Modeling and ASCE 7-16 Methods

**DOI:** 10.3390/s23042047

**Published:** 2023-02-11

**Authors:** Tabish Ali, Mohamed Nour Eldin, Waseem Haider

**Affiliations:** 1 Department of Civil, Architectural and Environmental System Engineering, Sungkyunkwan University, Suwon 16419, Republic of Korea; 2 Department of Electrical and Computer Engineering, Sungkyunkwan University, Suwon 16419, Republic of Korea

**Keywords:** soil-structure interaction, engineering demand parameters, finite element analysis, Machine Learning, Support Vector Machine, ASCE 7-16

## Abstract

Seismic design of structures taking into account the soil-structure interaction (SSI) methods is considered to be more efficient, cost effective, and safer then fixed-base designs, in most cases. Finite element methods that use direct equations to solve SSI problems are very popular, but the prices of the software are very high, and the analysis time is very long. Even though some low-cost and efficient software are available, the structures are mostly analyzed for the superstructure only, without using the geotechnical properties of the ground and its interaction effects. The reason is that a limited number of researchers have the knowledge of both geotechnical and structural engineering to model accurately the coupled soil-structure system. However, a cost-effective, less time-consuming and easy-to-implement technique is to analyze the structure along with ground properties using machine learning methods. The database techniques using machine learning are robust and provide reliable results. Thus, in this study, machine learning techniques, such as artificial neural networks and support vector machines are used to investigate the effect of soil-structure interactions on the seismic response of structures for different earthquake scenarios. Four frame structures are investigated by varying the soil and seismic properties. In addition, varying sample sizes and different optimization algorithms are used to obtain the best machine learning framework. The input parameters contain both soil and seismic properties, while the outputs consist of three engineering demand parameters. The network is trained using three and five-story buildings and tested on a three-story building with mass irregularity and a four-story building. Furthermore, the proposed method is compared with the dynamic responses obtained using fixed-base and ASCE 7-16 SSI methods. The proposed machine learning method showed better results compared with fixed-base and ASCE 7-16 methods with the nonlinear time history analysis results as a reference.

## 1. Introduction

Soil-structure interaction (SSI) is a very important phenomenon as it affects the time period and response of structures like the base shear, acceleration, and drift. The Mexico City and the Puebla earthquakes are evidence of this complex relationship [1]. Thus, the response of structures without considering soil domain may be misleading and may cause failure of the structure, depending on the site conditions [2,3]. Studies have shown that SSI can have a significant impact on the responses of structures to seismic excitations, amplifying story drifts, and accelerations [4,5,6]. Therefore, buildings may experience higher seismic demands when SSI is taken into account.

The inclusion of soil-structure interaction (SSI) in the analysis of structures on soft soils is crucial for accurate seismic assessment [7,8]. Additionally, the structures with extreme configurations are affected significantly by considering SSI. For example, a study on nuclear reactors showed that the soil properties had a very high impact on the seismic response of the reactor [9]. Fatahi et al. showed that, when SSI was considered, the performance level of structures was changed to near collapse from life safety [2]. Furthermore, another study showed that the design procedures without SSI considerations were not safe for structures on soil types De and Ee, which comprise stiff soils and soft clays [10]. Soil-structure interaction also increased the inter-story drifts for stiff soils and soft clays, making SSI investigation necessary for such soils [11,12]. Additionally, it was also shown that the damping of soil and structure had a great impact on the overall damping of the structure. The shear wave velocity and soil damping for soft soils have also been shown to have a significant effect on the maximum lateral displacement [13]. Past research also stressed that the variation of soil shear wave velocity, shear wave degradation ratio, structure-to-soil stiffness ratio, and structural aspect ratio, combined with the system stiffness, are the key parameters of a structural response [14]. Studies also showed that the SSI parameters have a key place in estimating the fragility of the foundations of a bridge and its abutment components like shear keys, span unseating, and bearings [15]. Other research showed that the cracking pattern of walls was mainly influenced by the soil-structure interaction [16]. On the other hand, the structures on some soil types showed smaller base shears for SSI designs compared with fixed-base designs. For instance, a study pointed out that there was no effect on the base shear of structures built on firm soils [17]. Similarly, the SSI effect decreased with the increase in the shear wave velocity of the soil [18]. Other research showed that the pile soil-structure interaction decreased the elastic-plastic inter-story drift compared with shear force and acceleration [19]. Therefore, to be on the safe side, the effect of SSI should be included in the analysis of the structures and then its inclusion or exclusion be decided for the final assessment.

The SSI designs are easily implemented using the finite element method (FEM) in commercial software. However, these are time consuming and complicated as they need more calculations and the software packages are also expensive. Additionally, a limited number of engineers have adequate knowledge of both structural and geotechnical designs. These challenges demand a low-cost, reliable, and easy-to-implement method. Recent studies have demonstrated the potential of machine learning (ML) in predicting structural response, including some that consider soil-structure interaction (SSI) [20,21,22]. An artificial neural network (ANN) is one of the ML techniques that is trained with past data and trends, so that afterwards it can predict the future responses [23,24,25,26]. Studies showed that the responses of bridges and buildings subjected to earthquakes were successfully and accurately predicted by the neural network approach [27,28,29,30,31]. It was also mentioned in some case studies that the ML methods were better in performance compared with conventional methods [32,33,34,35,36,37]. For example, the roof displacement, base shear and base bending moments were precisely predicted by the ANN model when compared with the finite element analysis (FEA) results [38]. Khatibinia et al. used a wavelet-weighted least squares support vector machine for seismic reliability assessment of reinforced concrete structures including SSI [11]. Farfani et al. used data-based methods to produce more experimental data for the seismic analysis of soil-pile-structure systems, and Mirhosseini used support vector regression to predict the seismic response of building systems considering SSI effects [21,22]. Other studies also attempted to predict building response without considering SSI using different machine learning techniques such as supervised and unsupervised learning algorithms, ANN, and convolutional neural networks [39,40,41]. Therefore, the machine learning techniques are accurate in predicting the structural responses and use less computational effort.

Researchers have investigated the seismic demands of structures considering SSI using conventional methods; however, they tend to rely on a single performance metric such as maximum inter-story drift or base shear, without considering multiple performance measures that are required for both serviceability and safety aspects [2,10]. Most studies focus on structural safety, with few considering the serviceability aspect, which is important for facilities such as hospitals and data centers [42]. Floor acceleration is commonly used as the representative engineering demand parameter for serviceability [43]. Another commonly overlooked aspect is the increase in seismic base shear demands on foundations due to global retrofitting [44,45,46]. Thus, the performance-based seismic design requires multiple engineering demand parameters for making the seismic fragility groups required for the assessment and design of buildings [47,48]. Furthermore, the recent seismic evaluation and retrofit standards require consideration of different limit states in the seismic assessment process and the prediction of the type of seismic enhancement required to meet a given performance level [49]. Therefore, there is a need for ML frameworks that consider both safety (maximum inter-story drift ratio, D, and base shear, V) and serviceability (floor acceleration, A) aspects in the seismic performance assessment.

Based on the previous discussion, it is clear that there is a need for comprehensive studies on the seismic performance assessment that considers different input variables related to the structure, soil-structure interaction, and earthquake events. The current research aims to address the lack of a seismic assessment ML framework that considers different engineering demand parameters (EDPs) and multiple limit states criteria, such as life safety and collapse prevention, while taking into account soil-structure interaction. The study develops a framework based on machine learning techniques for predicting the seismic performance of low-to-mid-rise frame structures considering SSI. The framework takes into account both safety (maximum inter-story drift ratio, D, and base shear, V) and serviceability (floor acceleration, A) aspects in the seismic performance assessment. The proposed framework is then compared to conventional methods that consider SSI, for example the ASCE 7-16, and verified using nonlinear time-history analysis. The proposed procedure provides an expert opinion on the structural seismic performance considering the variability in SSI, structural characteristics, and ground motion input, filling an important gap in the research area and providing a valuable insight for seismic assessment and design.

## 2. Methodology

The soil and structure are modeled concurrently using OpenSees, an open-source finite element software [50,51]. After making the main model in OpenSees with the help of TCL language, MATLAB is used for multiple runs [52,53]. The MATLAB is programmed to open the OpenSees software multiple times, and each time the input parameters are selected from a pre-defined matrix. Thus, the time-consuming database-making process is simplified.

### 2.1. Modeling the Superstructure (Building)

**Figure 1:**
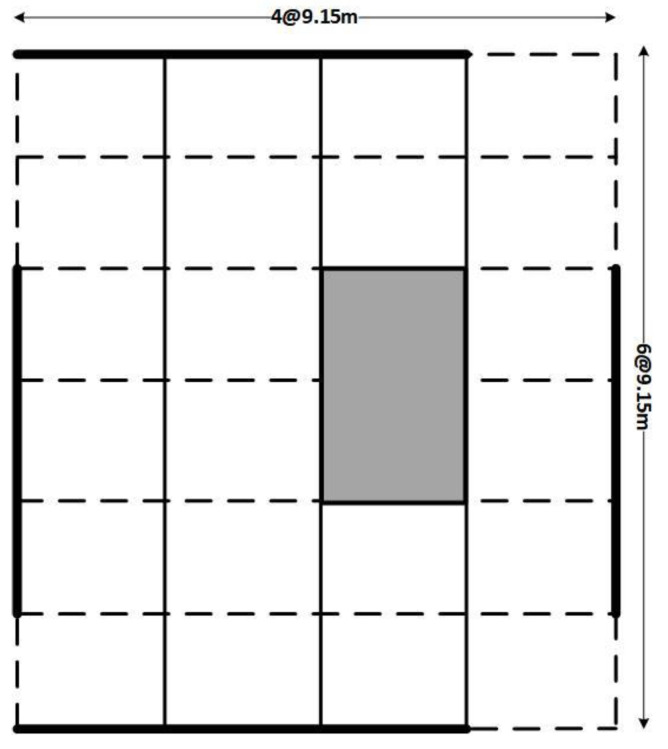
Top view of the structure.

**Figure 2:**
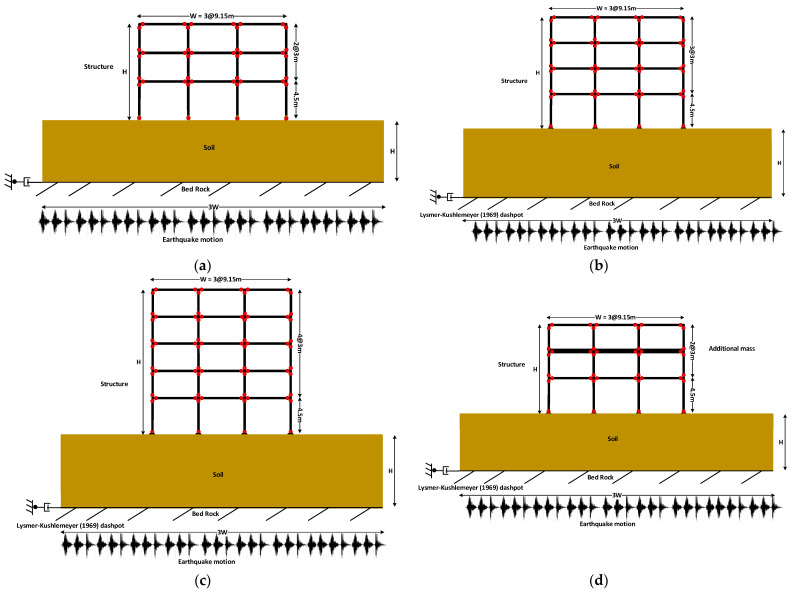
The frame structure models with the soil domain and ground motion. (**a**) Model of the three-story structure. (**b**) Model of the four-story structure. (**c**) Model of the five-story structure (**d**) Model of the three-story mass irregularity structure.

The top view and front elevation of the structures are shown in Figure 1. For simplicity, one of the outer frames in the short direction is used for analysis (ASCE-41 2017). Based on AISC-360 2016, the frames are designed for gravity loads of 4.1 kN/m^2^ and 2.5 kN/m^2^ of dead and live loads, respectively. The steel is assumed to have a nominal yield strength of 345 MPa and an elastic modulus of 200 GPa, with 0.3 poison ratio and 7850 kg/m^3^ density. Columns and beams are modeled as beam–column elements using an element library. The connection of columns and beams is carried out with the help of zero-length elements. The modified Ibarra–Krawinkler deterioration model is used for a nonlinear force–deformation relationship with the help of rotational springs [54]. The rotational springs connected to the elastic beam–column elements induce crack properties in the structure. The models are shown in Figure 2 for three-story, four-story, five-story and three-story mass irregularity (MI) structures, respectively. The time periods are 0.66 s, 0.75 s and 0.89 s for the SSI models, respectively. All the structures comprise three bays. The standard sections used for beams and columns are shown in Table 1.

### 2.2. Modeling the Substructure (Soil)

The direct method is used to model the massless soil domain in which the FEM model is developed and boundary conditions are enforced upon it. The width of the soil domain is taken as three times the width of the building, and depth is taken as equal to the height of the building. For a satisfactory analysis, the width of the soil model should be three times the length of the structure [55]. The soil mesh is created in such a way that the desired aspects of the propagating waves are captured in the analysis. The minimum vertical element size in the soil column is set by the horizontal size of the elements. The number of elements is determined by the thickness of the soil deposit. Thus, for n total elements there will be 2n + 2 total nodes. The bottom portion is fixed to behave as a rock and the sides are free. Isoperimetric four-node quadrilateral finite elements having two degrees of freedom per node are used to model the soil region. The plane strain condition is considered using the elastic-isotropic material model in OpenSees. Equal DOF command is used in both horizontal and vertical directions to connect the structure with the soil. Common nodes and suitable constraints are used to achieve equal displacements for the two domains. Lysmer–Kushlmeyer dashpots are introduced for the radiation damping and prevention of reflection of outward-propagating dilatational and shear waves back to the structure [56]. The mentioned dashpots are enough to satisfy the radiation damping conditions. A 5% damping ratio is applied as the typical soil damping conditions are in the range of 3% to 10% [57]. The range of elastic modulus is 478 to 210,000 kN/m^2^ and the range of Poisson’s ratio is 0.2 to 0.45.

### 2.3. Modeling of the Earthquakes

**Figure 3:**
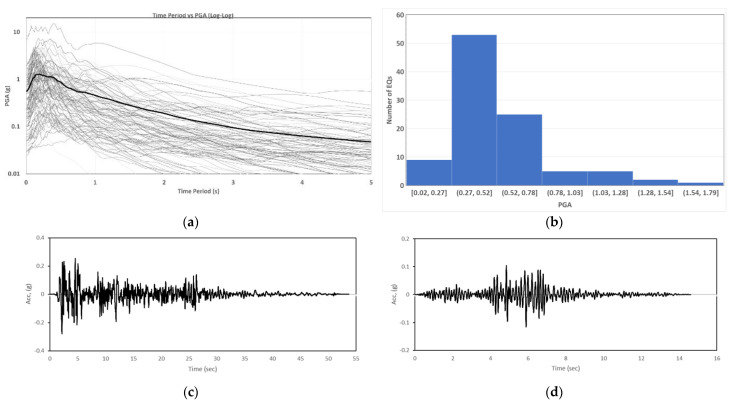
The earthquakes considered as input. (**a**) The response spectra of 100 input earthquakes. (**b**) The histogram of PGA ranges of the EQs. (**c**) The time history of Imperial Valley EQ. (**d**) The time history of Northern California EQ.

There are three important characteristics of the input ground motions that must be considered for earthquake modeling. First, the ground motions should be simple enough and readily available to the engineer to use them for design purposes. Second, the damage caused by the earthquakes must be a very close representation of the actual seismic events. Thirdly, they should portray the profile and intensity of the real earthquake. The intended framework of earthquakes in this study ensures these peculiarities. For instance, the inputs used in the current research are response spectra (frequency contents), and Peak ground acceleration (PGAs). These input data are considered to be a suitable representation of the earthquakes [33,41]. Another benefit is that the response spectra have the most influence on the nonlinear behavior of the ground excitations [33]. Thus, the machine learning techniques (ANNs and SVMs) will be robust in performance and excellent in predicting the nonlinear behavior, as the database considers important nonlinear properties in the input. In the present study a short version of the response spectrum extracted from the full versions is used. In this version, the controlling points at the natural periods of 0.01, 0.02, 0.05, 1, 1.5, 1.0 and 2.0 s are used as inputs to the machine learning framework. The earthquakes investigated are selected from the database of PEER [58] considering the actual ground motions records. The variability in PGA, magnitude (M_w_), site to source distance, source-fault mechanism, lowest useable frequency, and shear velocity (V_s30_) can be seen in Table 2. To introduce diversity in the input seismic events, 1000 ground motions records of magnitude range 3.5 to 8.5 have been picked from PEER. A total of 90% of the earthquakes had very little PGAs while only 10% of these had a very huge PGA. In this regard, a total of 100 earthquakes having a PGA range of 0.02 g to 1.79 g were selected, and a scale factor of 4 was used for these ground accelerations. For the range of 0.3 g to 0.6 g, being the practical range of seismic building design, a greater number of samples were used. The characteristics of the ground motion records used are listed in Table 2 with four sample earthquakes. The variability of the input ground motions over the expected range of occurrence is guaranteed from this selection. The EQs were applied at the bottom of the soil layer as a total base acceleration. Figure 3 is the representation of the response spectra and the histogram of the PGA range of the 100 earthquakes used as inputs. It also illustrates the time history of two sample EQs used in the NLTHA.

### 2.4. Database for Machine Learning Methods

**Figure 4:**
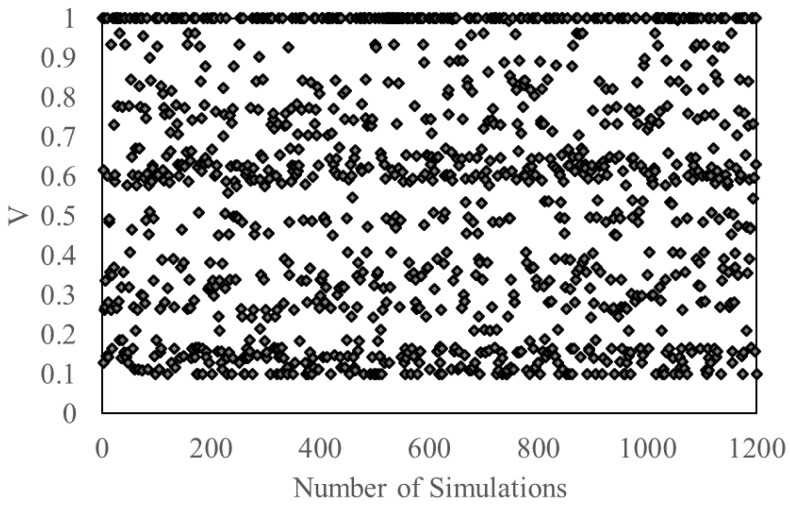
The normalized data spread showing the distribution of base shear.

In this section, the database used for the machine learning is discussed. After running around 1200 simulations for each structure type, the database containing inputs as spectral response, number of stories, Young’s modulus and Poisson’s ratio and a single output as base shear (V), maximum inter-story drift ratio (D), or acceleration (A) is developed with the help of a MATLAB script that automatically saves them in different files. These files are then combined in a single Excel sheet, which is then normalized to be used in the machine learning methods for training of ANNs and SVMs. Figure 4 shows the distribution of the normalized data over the number of simulations in the case of the base shear.

### 2.5. Implementing the Machine Learning Techniques

**Figure 5:**
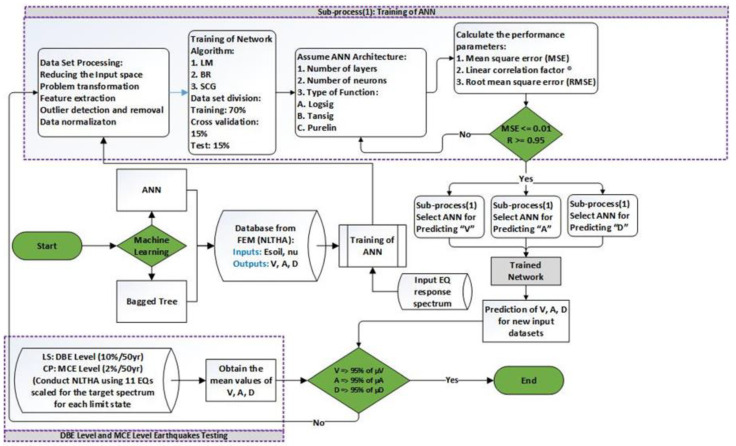
Flow chart of procedure adopted for machine learning.

The methodology used to apply the machine learning techniques is discussed in this chapter. Figure 5 shows a flow chart of the complete process. First, the machine learning method is decided then the database is established, which is obtained from FEM analysis to train the network, and then it is tested to predict the output. Before training, outliers were removed and the data were trimmed to be reasonable. Afterwards, different algorithms are used for training. A total of 70% of the data is used for training, 15% is used for cross validation, and 15% is used to test the data. The type of the algorithm, the number of layers, the number of neurons, and the type of the function are varied to achieve the best ANN architecture based on the mean square error (MSE) and the linear correlation coefficient (R) values. The selection criteria for MSE are less than or equal to 0.01 and for the R are greater than or equal to 0.95. Then, the trained network is tested with the help of 22 earthquakes considering maximum considered earthquake (MCE) and design-based earthquake (DBE). The ANN is trained on a three- and a five-story structure and tested on a four-story and a three-story mass irregularity structure.

## 3. Application of the Machine Learning techniques

### 3.1. Training and Test of the ANNs

**Figure 6:**
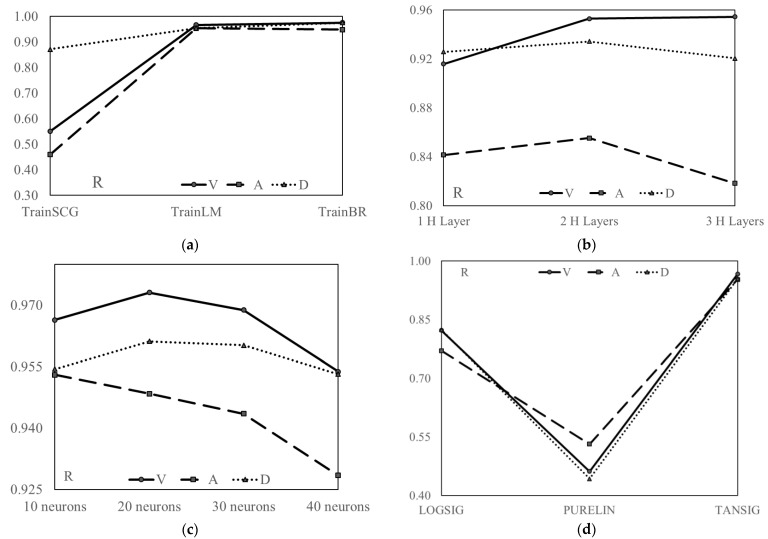
The effect of different parameters on the performance of the ANN. (**a**) Effect of the algorithm. (**b**) Effect of the number of layers. (**c**) Effect of number of neurons. (**d**) Effect of the type of function.

The ANNs were trained with different algorithms; such as the scaled conjugate gradient (TrainSCG), Levenberg–Marquardt (TrainLM) and Bayesian regularization (TrainBR) backpropagations, number of neurons, and number of layers and functions. Then, the effect of number of layers in the neural networks was analyzed. Afterwards, the effect of the number of neurons on the response of structures was determined. In the end, a comparison between the types of functions such as the log-sigmoid transfer (LOGSIG) function, pure linear transfer function (PURELIN), and hyperbolic tangent sigmoid transfer function (TANSIG), which are used for the training of the ANN, was performed. Figure 6 shows the results of the variation of parameters of the ANN. From the R values it is evident that TrainSCG has poor results compared with TrainBR and TrainLM. However, the latter two have almost the same results and in this study only TrainLM is used for future trainings. As far as the effect of the number of layers is concerned, for each of V, A and D, the trainings showed that a network with two hidden layers is the best for predicting the responses. The R values of the network with fewer than or greater than two hidden layers were poor in performance compared with those with two hidden layers. Finally, it is noted that 10 neurons were the optimum amount for accurate prediction, while increasing or decreasing the number of neurons is not recommended as this demonstrated poor results considering all V, A and D. As far as the type of the function is concerned, TANSIG performed better for predicting the structural dynamic responses while PURELIN had the lowest performance.

**Figure 7:**
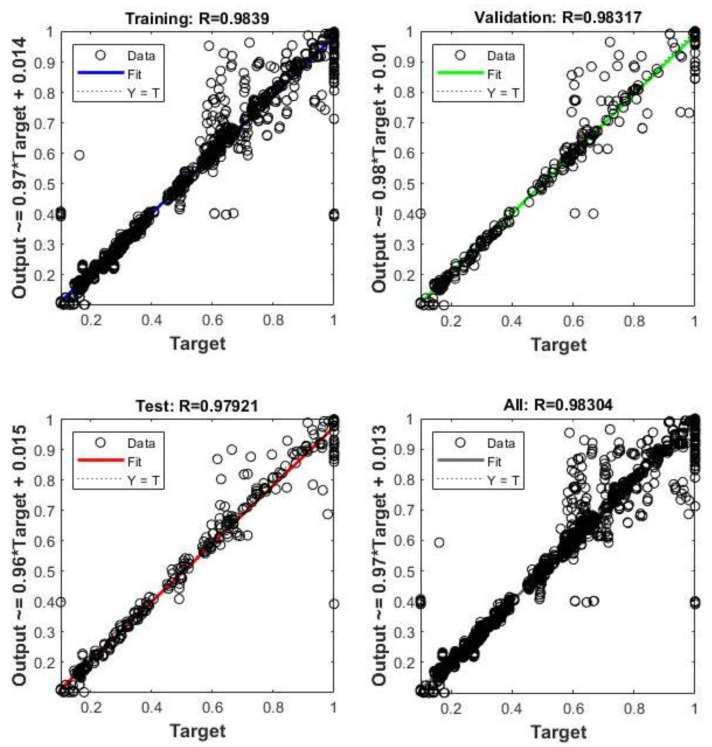
R values of the Training, Validation, and Test of ANN for base shear output.

**Figure 8:**
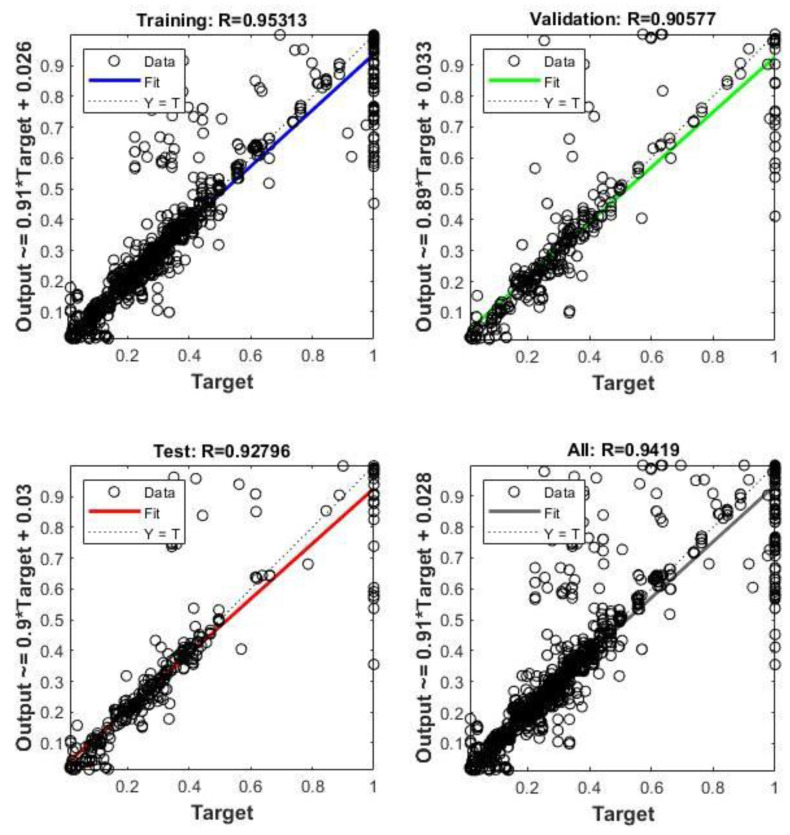
R values of the Training, Validation, and Test of ANN for acceleration output.

**Figure 9:**
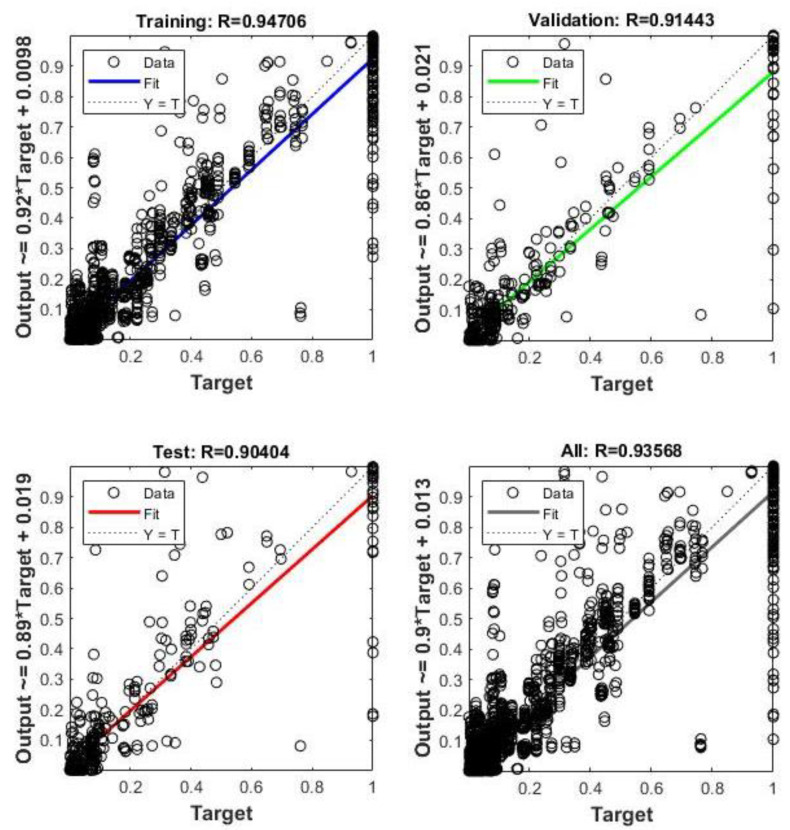
R values of the Training, Validation, and Test of ANN for drift output.

To conclude, a combination with the best results was selected and used for further comparisons and calculations. ANN with two hidden layers, 10 neurons each, TrainLM algorithm and TANSIG function showed the most accurate results. To demonstrate this statement further, the R values for each of Training, Validation, Test and All are shown in Figure 7, Figure 8 and Figure 9. The R value is greater than 96% in the case of base shear, 90% for acceleration, and 94% for drift predictions. 

### 3.2. Training and Test of the Support Vector Machines (SVMs)

**Figure 10:**
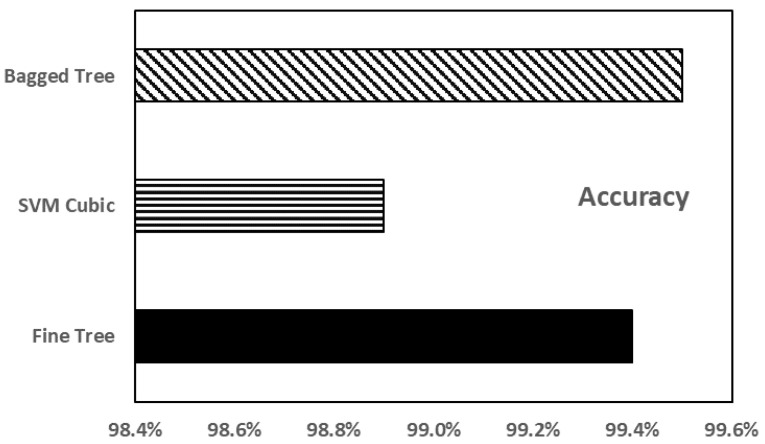
Accuracy of the different SVM models.

SVMs are trained after normalizing the data, as in the case of ANNs. Even though many SVM techniques were used for training, only three techniques with better results are mentioned here. These are fine tree, SVM cubic, and bagged tree. Figure 10 shows the comparison of the accuracies of these methods. It is observed that the bagged tree had the highest accuracy and the best prediction result of around 99.5%. Fine tree showed 99.4% accuracy whereas SVM cubic showed the least accuracy of about 98.9%.

### 3.3. Comparison of the Performance of the ML Techniques

**Figure 11:**
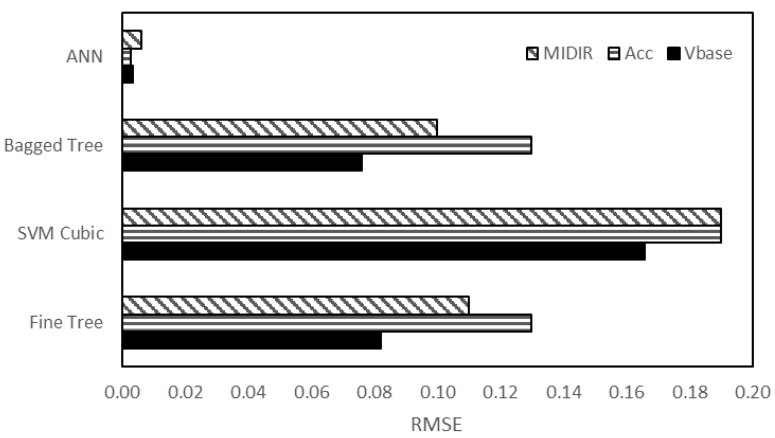
Comparison of RMSE values of SVMs and ANN.

After completing the training, validation and testing of the different machine learning techniques, the best of the best is selected as the proposed ML method. The comparison of these methods is shown in Figure 11. The root mean square error (RMSE) for base shear, acceleration, and MIDR is shown for each of fine tree, SVM cubic, bagged tree and ANN. It is seen that the ANN with around 0.01 value has the minimum RMSE for all the three responses of the structure. Thus, ANN is selected as the best method for predicting the dynamic response of structures coupled with soil.

## 4. ASCE 7-16 Methodology

**Figure 12:**
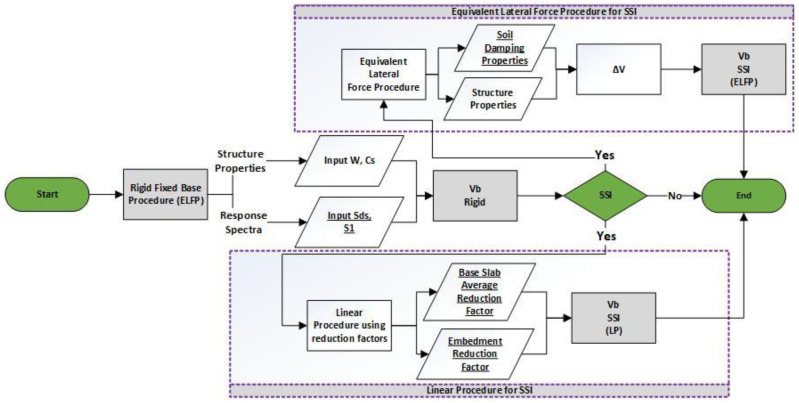
Flow chart of ASCE 7-16 procedure for SSI.

This section is about the approach adopted to obtain results from the ASCE method for the SSI analysis. The ASCE 7-16 is the latest version and very limited studies have been done. A recent study showed that both the ASCE 7-10 and ASCE 7-16 result in larger and similar structural responses compared with fixed-base design methods for structures with surface foundation. For structures on very soft soils, the new SSI provisions of ASCE 7-16 showed conservative designs [59]. It is revealed that the practicing SSI provisions resulted in nearly the same, higher, or lower level of risk as that of fixed-base design structures, which were categorized as optimal, excessively conservative, or unsafe designs. For structures on Site Class D, the SSI provisions yielded either unsafe or uneconomic designs. For structures on Site Class E, practicing SSI provisions of ASCE 7-16, in lieu of fixed-base regulations, might result in overly conservative designs [12]. It was concluded that both the National Earthquake Hazards Reduction Program (NEHRP) and the current provisions resulted in unsafe designs for structures with surface foundations on moderately soft soils. For structures on very soft soils, the method of NEHRP was more conservative [60]. Thus, there is a lot of room to criticize the current ASCE provision. The flow chart in Figure 12 is an illustration of the steps taken to obtain the responses from this method. In the ASCE fixed-base method, the soil effect is ignored whereas, in both the equivalent lateral force procedure and nonlinear procedure, the effect of soil is introduced with the help of different factors that depict the soil properties.

## 5. Results and Discussion

**Figure 13:**
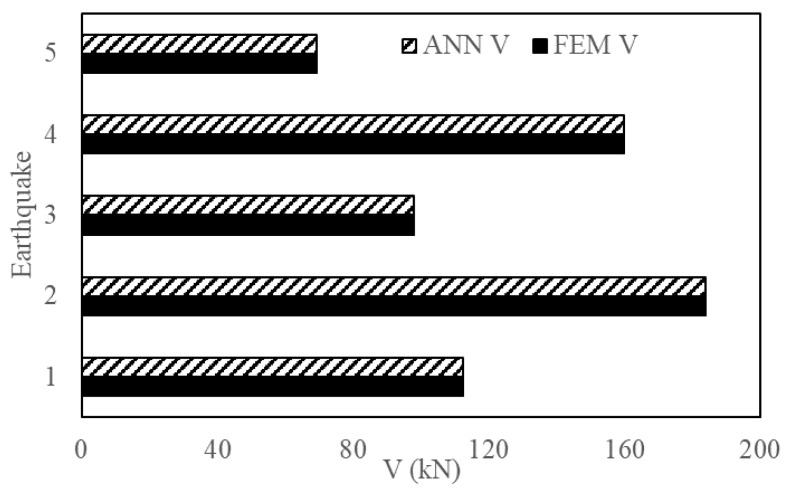
Base shear of a four-story structure obtained from ANN and NLTHA for five EQs.

**Figure 14:**
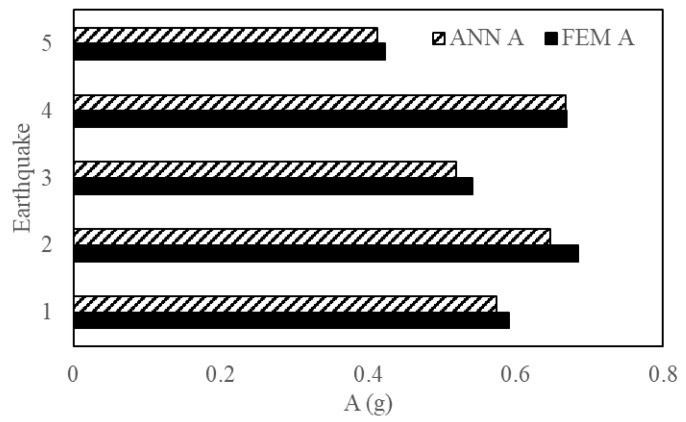
Acceleration of a four-story structure obtained from ANN and NLTHA for five EQs.

**Figure 15:**
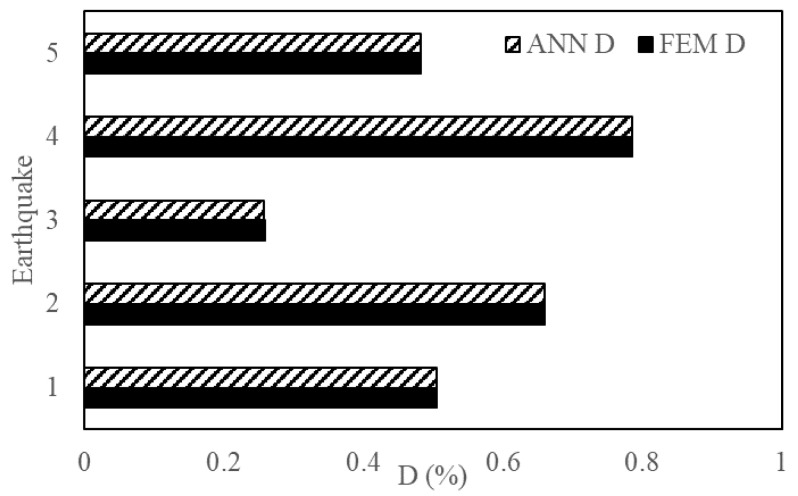
MIDR of a four-story structure obtained from ANN and NLTHA for five EQs.

This section reports the performance and accuracy of the proposed method. The generalization potential of the proposed method was examined by introducing new EQs to test the ANN framework. The characteristics of these EQs are given in Table 3. The ML results are validated with the FEM simulation results. Figure 13, Figure 14 and Figure 15 show the output engineering demand parameters (base shear, acceleration, and maximum inter-story drift) of a four-story structure predicted by the ANN, which was based on three- and five-story structures. For the selected earthquakes, the ANN framework showed accurate results when compared with the NLTHA.

**Figure 16:**
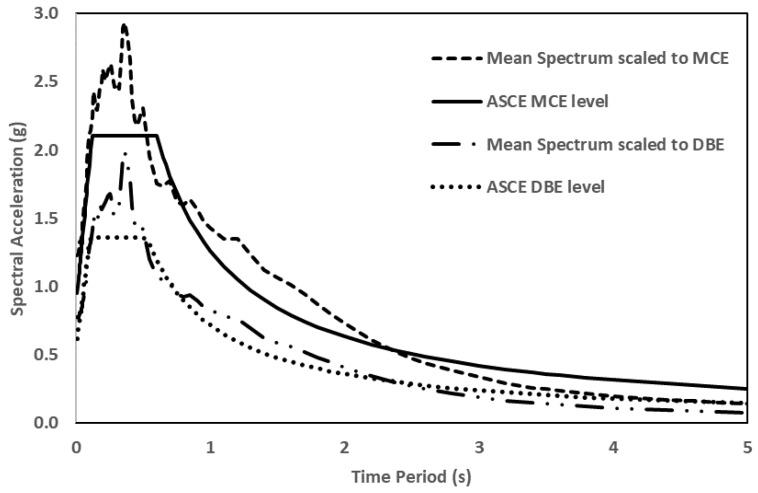
Spectrum of MCE, DBE and their means used to test a three story mass irregularity structure.

Similarly, the ANN model is also tested on a three-story mass irregularity structure. The earthquakes, this time, consisted of design-based EQs (DBE) and maximum considered EQs (MCE) to achieve meaningful results and to compare them with the results obtained from other conventional methods. Table 4 lists the characteristics of these EQs. Figure 16 shows the design spectrum of these EQs.

**Figure 17:**
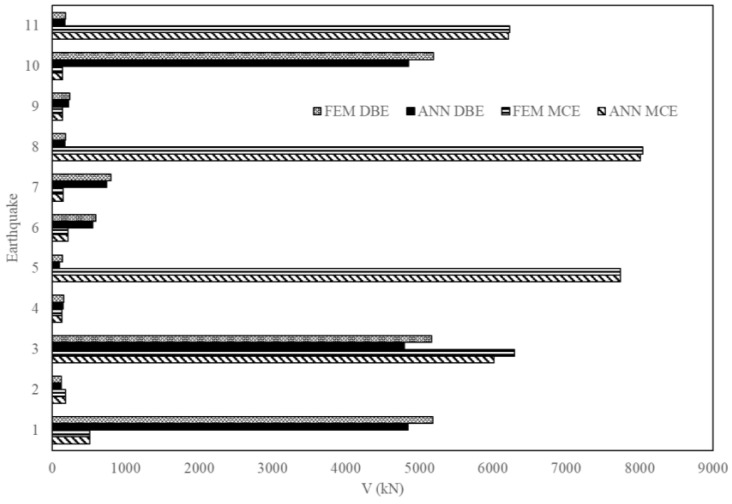
Base shear of a three-story mass irregularity structure obtained from ANN and NLTHA considering DBE and MCE levels.

**Figure 18:**
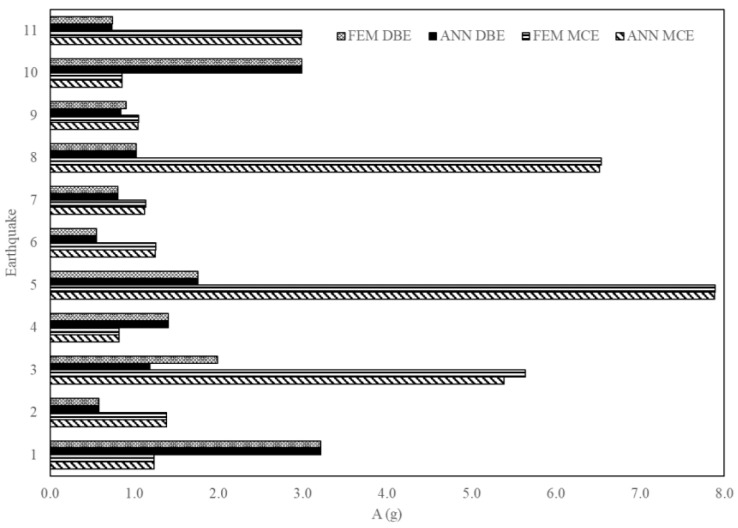
Acceleration of a three-story mass irregularity structure obtained from ANN and NLTHA considering DBE and MCE levels.

**Figure 19:**
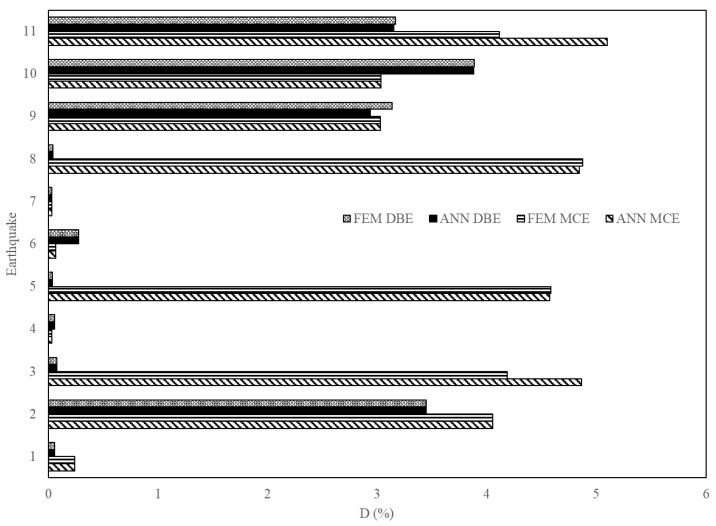
MIDR of a three-story mass irregularity structure obtained from ANN and NLTHA considering DBE and MCE levels.

Figure 17, Figure 18 and Figure 19 are a comparison of the V, A and D of NLTHA and ANN obtained for the three-story mass irregularity structure. The ANN achieved a good prediction for the 11 earthquakes of MCE and DBE levels. This shows the robustness of the proposed method.

**Figure 20:**
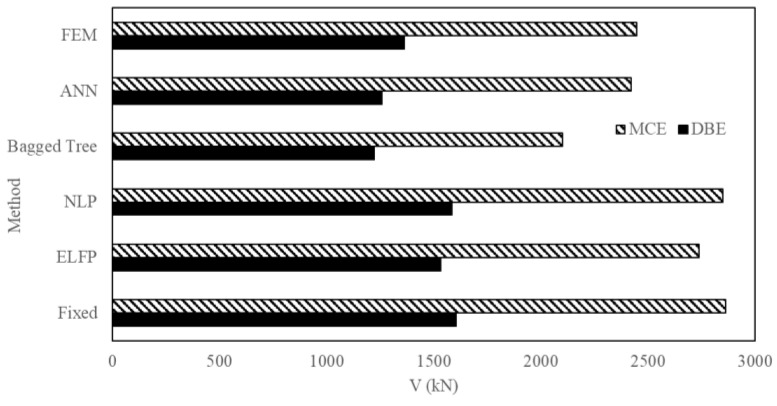
Comparison of base shear obtained from different techniques.

**Figure 21:**
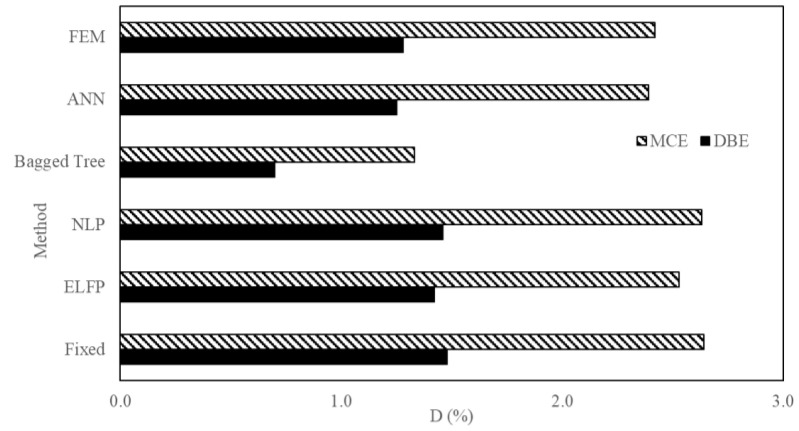
Comparison of MIDR obtained from different techniques.

Then the results of the proposed method were compared with those of the conventional methods for SSI seismic response. The FEM is again taken as reference for this comparison. Figure 20 and Figure 21 are the comparison of V and D values from ANN, SVM, and ASCE 7-16 methods. It is noted that ANN is the closest to the FEM (NLTHA) outputs compared with other methods. Thus, ANN is the most reliable method among the techniques discussed. Table 5 and Table 6 show the quantitative results. ANN predicted approximately the same as the FEM results, whereas the fixed-base method showed higher values compared with the equivalent lateral force procedure (ELFP) and ML techniques. ELFP showed results close to FEM compared with the bagged tree (SVM) method but the values were higher than the FEM method. The error percentage for ANN was less than 2% for MCE and below 8% for DBE with the FEM (NLTHA) as a reference. The proposed method is superior over the conventional techniques. For example, Table 6 shows, in the case of MIDR, ELFP overestimated the results by 10.94% and 4.54% for the DBE and MCE levels, respectively. In the fixed case, these values increased to 15.63% and 9.09%, respectively, and in the NLP case they increased to 14.10% and 8.68%, respectively. However, in the ANN case, the difference was reduced to 0.03% and 1.23% for the DBE and MCE levels, respectively. In the case of V, the ELFP overestimated the results by 12.53% and 11.89%, respectively. Other ASCE methods showed more conservative results than the ELFP. However, the differences in the case of the ANN were reduced to 7.75% and 0.96% for the DBE and MCE levels, respectively. This shows that the proposed framework provides a higher accuracy in predicting V and D compared with conventional ASCE methods. It was found that the bagged tree technique underestimates the response values in general.

In summary, the proposed method has various advantages over the conventional techniques. For example, the proposed method considers multiple EDPs, which are essential for seismic assessment at multi hazard levels. The D, A and V values provide insight for retrofitting. A higher D value means the structure should be stiffened. If V is high, the material strength should be increased, and huge A values warn about the safety of non-structural elements. Furthermore, this method is also applicable to complex structures like mass-irregularity buildings. An interesting finding is that the ML-based methods showed less conservative but more accurate results compared with ASCE methods with NLTHA as a reference. One reason behind this is that the ML framework is trained on the NLTHA data. The other reason is the practical limitations in the ASCE method, for example, the conservative design approach for safety, simplified models, and assumptions for SSI consideration.

## 6. Conclusions

This study presented a machine-learning-based framework to predict the seismic response of structures considering the effect of soil-structure interaction. The machine learning inputs were prepared taking into account the soil, seismic, and structure properties. The outputs consisted of multiple engineering demand parameters such as the base shear, acceleration, and maximum inter-story drift. These are essential for probability-based seismic design, which considers serviceability, strength, and safety of the structure. The database was trained with a three- and a five-story structure and tested on a four-story and a three-story mass irregularity structure. The soil domain was modeled as a continuum for better soil-structure interaction effects. The results point out the artificial-neural-network-based model is the most accurate, fast, reliable, and easy to implement method to obtain the seismic response of structures. The main outcomes of the study are summarized as follows:

- The machine learning framework achieved more than 95% accuracy with two layers having ten neurons each, TANSIG function and TrainLM algorithm.
- The soil-structure-interaction-based artificial neural network model results were in good agreement with those of the nonlinear time history analysis compared with fixed-base, support vector machine and ASCE 7-16 linear soil-structure interaction methods.
- The errors in artificial neural network predictions were less than 2% for the maximum considered earthquake and below 8% for the design-based earthquake with nonlinear time history analysis as a reference.
- One of the interesting finding is that the artificial neural network framework provided higher accuracy in predicting base shear and drift compared with conventional ASCE methods.
- The proposed framework showed high generalization potential for the range of low-to-mid-rise frame structures. It also successfully predicted the behavior of mass irregularity structures.

For future research, an extensive database comprising complex structures with more stories and especially high-rise buildings along with sophisticated soil models is to be made and the machine learning framework be trained for even better and reliable results. In addition, for ASCE 7-16 comparison, other nonlinear procedures will be considered that are not included in this study. Different frame types and other structural variations will also be added to extend the current study.

## Figures and Tables

**Table 1:** Details of the cross-sections of the structures.

Elements	Model	Standard Section
Beams	3, 4, 5 story	W33 × 118
Columns	3 story	(0–3 story) W14 × 257
	4 story	(0–2 story) W14 × 311 (2–4 story) W14 × 257
	5 story	(0–2 story) W14 × 311 (2–5 story) W14 × 257

**Table 2:** Characteristics of the samples from 100 EQs used in NLTHA.

	Limit/EQ Name	PGA (g)	Magnitude (M_w_)	Source to Site Distance (km)	V_s30_ (m/s)	Lowest Useable Frequency (Hz)	Source-Fault Mechanism
Limits of parameters	Upper	1.800	7.62	218.13	1428.14	3.750	Normal; Reverse: Reverse Oblique; Strike Slip
	Lower	0.017	4.20	0.56	169.84	0.025	
Earthquake samples	“Ancona-06_Italy”	0.740	4.30	11.18	448.77	1.125	Normal
	“Golden Gate Park”	0.340	5.28	11.02	874.72	0.875	Reverse
	“Yorba Linda”	0.320	4.26	16.19	384.44	0.390	Strike Slip
	“Santa Barbara”	0.287	5.92	27.42	465.51	0.250	Reverse Oblique

**Table 3:** Characteristics EQs used to test a four-story building.

	Limit/ EQ Name	PGA (g)	Magnitude (M_w_)	Source to Site Distance (km)	V_s30_ (m/s)	Lowest Useable Frequency (Hz)	Source-Fault Mechanism
Limits of parameters	Upper	0.6447	6.61	63.34	529.09	0.625	Normal; Reverse: Reverse Oblique; Strike Slip
	Lower	0.3016	5.30	22.77	198.77	0.100	
Earthquake samples	“Northwest Calif-03”	0.3016	5.80	53.73	219.31	0.500	Strike Slip
	“Central Calif-01”	0.3409	5.30	25.81	198.77	0.375	Strike Slip
	“Parkfield”	0.3702	6.19	63.34	493.50	0.625	Strike Slip
	“San Fernando”	0.5797	6.61	22.77	316.46	0.100	Reverse
	“San Fernando”	0.6447	6.61	35.54	529.09	0.250	Reverse

**Table 4:** Characteristics of design-based and maximum considered test EQs for a three-story mass irregular structure.

	Limit/EQ Name	PGA (g)		Scale		Magnitude (M_w_)	Source to Site Distance (km)	V_s30_ (m/s)	Lowest Useable Frequency (Hz)	Source-Fault Mechanism
		DBE	MCE	DBE	MCE					
Limits of parameters	Upper	1.5506	2.107	4.9804	8.5508	7.36	114.62	527.92	0.375	Reverse, Strike Slip
	Lower	0.038	0.655	2.0934	1.3011	5.20	3.510	213.44	0.1	
Earthquake samples	“Imperial Valley-02”	0.5878	1.009	2.0933	3.5940	6.95	6.09	213.44	0.25	Strike Slip
	“Kern County”	0.6078	1.043	3.8256	6.5682	7.36	114.62	316.46	0.125	Reverse
	“Northern Calif-03”	0.3818	0.655	2.3369	4.0123	6.5	26.72	219.31	0.125	Strike Slip
	“Parkfield”	1.2277	2.107	2.7664	4.7497	6.19	9.58	289.56	0.1625	Strike Slip
	“Parkfield”	1.5506	0.890	4.3493	6.7153	6.19	15.96	527.92	0.1875	Strike Slip
	“Borrego Mtn”	0.5189	0.993	3.9113	4.4218	6.63	45.12	213.44	0.1	Strike Slip
	“San Fernando”	0.5788	1.289	2.5754	8.5169	6.61	22.77	316.46	0.1	Reverse
	“San Fernando”	0.7511	1.586	4.9606	1.3011	6.61	22.23	425.34	0.15	Reverse
	“San Fernando”	0.5580	0.958	4.9804	8.5507	6.61	24.16	452.86	0.1875	Reverse
	“Managua_Nicaragua-01”	0.8867	1.522	2.3847	4.0944	6.24	3.51	288.77	0.375	Strike Slip
	“Managua_Nicaragua-02”	0.7741	1.329	2.9452	5.0566	5.20	4.33	288.77	0.125	Strike Slip

**Table 5:** Values of the base shear obtained from different techniques for the three-story mass irregularity structure.

10%/50 yr (DBE)				2%/50 yr (MCE)		
Method	Vbase (kN)	Diff. from FEM	% Error	Vbase (kN)	Diff. from FEM	% Error
Fixed	1604.24	240.99	17.68	2864.2	416.477	17.01
ELFP	1534.00	170.75	12.53	2738.77	291.05	11.89
NLP	1585.65	222.40	16.31	2851.38	403.66	16.49
Bagged Tree	1222.73	140.52	10.31	2100.55	347.17	14.18
ANN *	1257.53	105.72	7.75	2424.01	23.71	0.96
FEM (reference)	1363.25	-	-	2447.72	-	-

* Best technique.

**Table 6:** Values of MIDR obtained from different techniques for the three-story mass irregularity structure.

10%/50 yr (DBE)				2%/50 yr (MCE)		
Method	MIDR (%)	Diff. from FEM	% Error	MIDR (%)	Diff. from FEM	% Error
Fixed	1.48	0.20	15.63	2.64	0.22	9.09
ELFP	1.42	0.14	10.94	2.53	0.11	4.54
NLP	1.46	0.18	14.10	2.63	0.21	8.68
Bagged Tree	0.70	0.58	45.31	1.33	1.09	45.04
ANN *	1.25	0.03	2.34	2.39	0.03	1.23
FEM (reference)	1.28	-	-	2.42	-	-

* Best technique.

## Data Availability

Not applicable.
